# The role of the microbiome in rheumatoid arthritis: a review

**DOI:** 10.1093/rap/rkad034

**Published:** 2023-04-03

**Authors:** Maria-Nefeli Tsetseri, Alan J Silman, David J Keene, Stephanie G Dakin

**Affiliations:** Nuffield Department of Orthopaedics, Rheumatology and Musculoskeletal Sciences, University of Oxford, Oxford, UK; Nuffield Department of Orthopaedics, Rheumatology and Musculoskeletal Sciences, University of Oxford, Oxford, UK; Nuffield Department of Orthopaedics, Rheumatology and Musculoskeletal Sciences, University of Oxford, Oxford, UK; Faculty of Health and Life Sciences, University of Exeter, Exeter, UK; Nuffield Department of Orthopaedics, Rheumatology and Musculoskeletal Sciences, University of Oxford, Oxford, UK

**Keywords:** RA, microbiome, dysbiosis, intestinal, pathogenesis

## Abstract

The close bidirectional relationship between the microbiome and the immune system is well supported, and a role of gut dysbiosis has been implied in many systemic autoimmune diseases. This review aims to provide a critical summary and appraisal of 6 murine studies and 16 clinical studies. The findings of the literature review suggest that gut dysbiosis precedes arthritis and that local intestinal inflammation leads to systemic inflammation in genetically predisposed individuals. However, the exact mechanism by which microorganisms provoke immune responses at distal sites remains to be elucidated. Although a characteristic RA microbiome was not identified, there were some common findings among studies: overabundance of *Prevotella copri* in early RA patients, and proliferation of the genus *Collinsela* and some *Lactobacillus* species. Three mechanisms by which microbiota might contribute to RA pathogenesis were proposed: inflammatory responses (*P. copri* and *Lactobacillus*), molecular mimicry (*P. copri*) and loss of intestinal barrier integrity (*Collinsella*). Larger longitudinal studies are required in order to shed light on the mechanisms involved and unravel the therapeutic potential of the microbiome, and clinical trials are needed to evaluate the safety and efficacy of the implied therapeutic interventions.

Key messages

- The findings of the literature review suggest that gut dysbiosis precedes arthritis and that local intestinal inflammation leads to systemic inflammation in genetically predisposed individuals.
- A characteristic RA microbiome was not identified, but there were some common findings among studies: overabundance of *Prevotella copri* in early RA patients, and proliferation of the genus *Collinsela* and some *Lactobacillus* species.
- Three mechanisms by which microbiota might contribute to RA pathogenesis are proposed: inflammatory responses, molecular mimicry and loss of intestinal barrier integrity.

## Introduction

### The microbiome and the host

The human body harbours a diverse population of microorganisms (bacterial, viral, fungal and archaeal) that reside at mucosal surfaces, including the gastrointestinal, genitourinary and respiratory tracts and the skin [1]. The collection of these microorganisms, their genes and genomes is known as the microbiome [2]. The collection of genes of a human microbial community, which is hundreds of times larger than the human genome, encode millions of proteins that work as an extension of our own genome and perform various metabolic, endocrinological and immunological functions [3]. The gastrointestinal tract, which has the largest mucosal surface, hosts the largest and most diverse microbial community [4, 5]. Up to 5000 species are estimated to colonize the gastrointestinal tract, predominantly bacteria belonging to the phyla Firmicutes and Bacteroidetes, while bacteria from Proteobacteria, Actinobacteria, Fusobacteria and Verrucomicrobia phyla are found in lower proportions [6, 7]. The microbiome varies greatly among individuals because it is affected by many factors, including vaginal delivery, diet and faecal microbiota transplantation [8, 9]. It also varies within the same individual regionally, according to the local environment, or temporarily, owing to exposure to environmental factors such as the use of antibiotics [9, 10].

### The microbiome and the immune system

The gut microbiota chiefly maintains a symbiotic relationship with the host and is essential for homeostasis and health. Symbiotic gut microbes play a central role in digestion and absorption of nutrients, in protection from pathogenic microbes and in the development and function of the immune system [7]. The relationship between the gut microbiome and the immune system has been implied since the 1960s, with studies on germ-free (GF) mice demonstrating an essential role of the microbiota in the structure and function of gut-associated lymphoid tissue [5, 11–14]. More recent studies are also underlining the importance of gut microbiota in the development of the intestinal immune system by showing that GF mice and gnotobiotic mice (colonized with a defined bacterial species) present various immune deficiencies, such as fewer lymph nodes, Peyer’s patches and Th17 cells, in addition to impaired Treg [3, 15–17]. Evidence showing that the gut microbiota has an effect on the quantity and function of circulating Th17 and Treg indicates that changes in the microbiome might precede systemic immunological responses [3, 16, 18]. Inversely, changes in the immune system lead to alterations of the composition and function of the gut microbiota, implying a bidirectional and dynamic relationship between the microbiome and the immune system [3].

### The microbiome and (inflammatory joint) disease

The reciprocal relationship described above is crucial in maintaining health, and any disruption can lead to disease. Lederberg *et al.* (2000) [19] were the first to speculate on the role of the microbiome in disease, and recent advances in omics technologies and bioinformatic analyses are currently establishing significant associations between the gut microbiome and a wide range of diseases, including gastrointestinal, metabolic, cardiovascular and rheumatic diseases [20]. Taking into consideration the robust epidemiological and molecular evidence that the microbiome determines the development and regulation of the immune system, it is not surprising that it is hypothesized that autoimmune disorders might be triggered by gut dysbiosis [2, 3]. The microbiome, via direct contact at the mucosal surfaces and also indirectly via the production of metabolites, might be a crucial initiator of autoimmune diseases [20]. Advances in high-throughput sequencing (16S rRNA and shotgun metagenomic sequences) has enabled us to study microorganisms that are unculturable, to identify bacterial species and to evaluate bacterial diversity. The human microbiome project and the MetaHIT project have produced large genomic databases (>70 million 16S rRNA gene sequences have been produced) accounting for multiple covariates and greatly expanding the potential for future microbiome research [21]. Together with advances in bioinformatics that allow analysis of these sequence data, our understanding of how microorganisms function, interact with their environment and vary among individuals has broadened and, in fact, a correlation between autoimmune diseases and the composition and function of the microbiome is being established. Research using GF mice has also provided causal evidence that the microbiome contributes to the pathogenesis of autoimmune diseases [20]. The recent technological advances have led to the accumulation of a growing body of evidence focusing on the role of the microbiome in inflammatory joint diseases: most notably in RA, PsA and AS [22]. This review focuses on the role of the intestinal microbiome in the pathogenesis of RA by critically presenting and analysing available data from murine and human studies in order to reveal the therapeutic prospects of the microbiome in the management and prevention of rheumatic disease.

### The microbiome and RA

RA is a chronic systemic autoimmune inflammatory disease characterized mainly by joint pain and inflammation leading to progressive disability [23]. It is one of the most common autoimmune diseases, affecting ∼1% of the population worldwide, and is associated with multiple co-morbidities, including cardiovascular, pulmonary, psychological and musculoskeletal disorders [23]. The aetiology of RA is not completely understood; however, it is known that a complex interaction between genetic and environmental factors induces a pathological activation of the immune system, leading to the clinical onset of RA [23]. The HLA-DRB1 allele, one of the strongest genetic risk factors for RA, has been suggested to promote autoreactive immune responses by shaping T-cell repertoire selection, by antigen presentation and/or by peptide affinity alteration [23]. Other genetic immunoregulatory markers have also been identified to increase susceptibility to RA [23]. A study on twins has shown a concordance rate of ∼15% for RA in monozygotic twins, implying that environmental factors, such as smoking, diet and infections, are involved in the development of disease [24]. Although infections have been associated with RA for more than a century, and antimicrobial drugs have been shown to have therapeutic effects in RA patients, a single infectious agent has not been identified, and the mechanism is not fully understood [2, 3].

The development of cutting-edge DNA sequencing techniques has allowed the taxonomic and functional characterization of microbiota and has renewed interest in the role of microbes in the pathogenesis of autoimmune diseases, such as RA [3]. Immunological findings in RA patients include alterations in the quantity and function of T and B cells, imbalances between Th17 and Treg, overproduction of TNF-α, increased pro-inflammatory cytokines and the presence of ACPAs [5]. ACPAs are an important biomarker because they are detectable before the clinical onset of RA, and they are also associated with disease severity [2]. Increasing evidence suggests that these immune abnormalities might occur at the mucosal level, owing to a high load of microbial antigens, and implies a causal role of the microbiome in the pathogenesis of RA [3, 25]. Two oral mucosa citrullination-promoting bacteria (*Porphyromonas gingivalis* and *Aggregatibacter actinomycetemcomitans*) have been identified, potentially explaining the epidemiological associations between periodontitis and RA [25]. Furthermore, murine models of arthritis are showing increased numbers of T cells and pro-inflammatory cytokines in the intestinal mucosa [25], and alterations in the gut microbiota of early and established RA patients have been well reported [3].

Nonetheless, research on the mechanisms by which the microbiome might affect local and systemic immune regulation and contribute to the development of autoimmune inflammatory diseases is limited. In this review, we critically present the available data from murine models of arthritis to address the pathogenetic role of the microbiome in the development of inflammatory joint diseases, then we analyse clinical evidence from human RA patients and present the suggested molecular mechanism by which the microbiome contributes to arthritis. The role of the microbiome as a diagnostic and prognostic biomarker is also be discussed. Lastly, the therapeutic and preventive potential of microbiome manipulation through diet, probiotic supplementation and faecal microbiota transplantation is reviewed briefly.

## Methods

PubMed was searched on 14 January 2021, using free-text words to search the title or abstract fields and relevant indexing to retrieve references about the role of the microbiome in inflammatory joint disease. The results were limited to those published in the English language from 2010 to the search date. The full search strategy for PubMed is available in Supplementary Data S1, available at *Rheumatology Advances in Practice* online. The reference lists of included papers were assessed, and forwards citation searching was conducted in Google Scholar to check for additional relevant studies. Six papers on murine models of arthritis and 16 papers on human patients were identified (see Table 1 and Supplementary Data S1, available at *Rheumatology Advances in Practice* online).

**Table 1.** Murine models of arthritis and results

Author	Murine model	Results
Maeda *et al.* (2016) [27]	SKG	GF SKG: ↓ arthritis incidence and severity
		*Prevotella* dominated RA SKG: ↑ dendritic cell stimulation ↑ Th17 ↑ arthritis incidence and severity
		*Prevotella copri* monocolinization: ↑ Th17, IL-23 and IL-17 ↑ arthritis incidence and severity
Rehaume *et al.* (2014) [28]	SKG	GF SKG: ↓ arthritis incidence and severity
		SPF SKG: ↑ arthritis incidence ↑ Th17, IL-23 and IL-17 Ileitis
		Schaedler flora SKG: ↑ arthritis incidence Enthesitis Ileitis
Rogier *et al.* (2017) [30]	IL-1ra^−/−^	IL-1ra^−/−^: ↓ microbiota diversity ↑ *Helicobacter* genus ↓ *Prevotella* genus
		Germ-free IL-1ra^−/−^: ↓ arthritis incidence and severity ↓ Th17
		SFB IL-1ra^−/−^: ↑ arthritis incidence
		Tobramycin treatment: ↓ arthritis severity ↓ *Helicobacter* genus
Wu *et al.* (2010) [32]	K/BxN	Germ-free K/BxN: ↓ disease incidence and severity ↓ autoantibodies (IgG, IgG1)
		SFB K/BxN: ↑ disease onset and severity ↑ Th17
Jubair *et al.* (2018) [33]	CIA	CIA: ↓ diversity ↓ *Bacteroidetes* phylum ↑ Lactobacillaceae family ↑ Lachnospiraceae family Mucosal barrier impairment Intestinal mucosal inflammation
		GF CIA: ↓ disease incidence and severity ↓ inflammatory cytokines (IFN-γ, IL-1β, IL-6 and TNF-α) ↑ IL-17A, IL-22 and IL-23 (intestine)
Liu *et al.* (2016) [34]	CIA	*Lactobacillus salivarius* and *Lactobacillus plantarum* CIA: ↓ disease incidence and severity ↓ inflammatory cytokines (IL-17, TNF-α) ↑ IL-10 ↓ Th17 ↑ Treg

CIA: collagen-induced arthritis; GF: germ free;  SFB: segmented filamentous bacteria.

## Results and Discussion

### Murine models

Several experimental studies on murine arthritis have investigated the causal relationship between the intestinal microbiome and the pathogenesis of arthritis. This review identified and critically presents four types of models: SKG model, IL-1 receptor antagonist knockout (IL-1ra^−/−^), K/BxN and collagen-induced arthritis (CIA) (see Table 1).

#### SKG model

SKG mice bear a point mutation in the *Zap70* gene and spontaneously develop Th17 cell-dependent arthritis [26]. Interestingly, SKG mice reared in GF conditions did not develop arthritis [27, 28]. However, inoculation of specific microbes was sufficient to induce arthritis. Maeda *et al.* [27] demonstrated that colonization of SKG mice with human *Prevotella*-dominated microbiota from RA patients led to stimulation of intestinal dendritic cells, increased production of Th17 cytokines in the large intestine, and rapid induction of arthritis. In addition, monocolonization of *Prevotella copri* also induced Th17 cell responses and arthritis in SKG mice. Rehaume *et al.* (2014) [28] showed that recolonization with Schaedler flora of GF SKG mice increased the incidence of arthritis, and that ileitis is microbiota dependent.

#### IL-1ra^−/−^ model

IL-1ra^−/−^ mice spontaneously develop autoimmune T cell-mediated arthritis [29, 30]. Rogier *et al.* (2017) [30] found that IL-1ra^−/−^ mice had decreased bacterial diversity and altered composition (increased *Helicobacter* species, low *Ruminococcus* species), in addition to an increased Th17 population. GF IL-1ra^−/−^ mice did not develop arthritis. This finding is in agreement with a previous study on IL-1ra^−/−^ mice [29], which also showed that *Lactobacillus bifidus* monocolonization led to rapid induction of arthritis [29]. Tobramycin treatment was shown to diminish arthritis significantly and eliminate *Helicobacter* species [30].

#### K/BxN model

K/BxN mice possess a transgenic T-cell receptor and develop spontaneous arthritis, owing to the production of autoantibodies against glucose-6-phosphate isomerase [31]. Wu *et al.* (2010) [32] showed that in GF conditions, autoimmune arthritis does not develop and the number of Th17 is significantly reduced, whereas inoculation with segmented filamentous bacteria is sufficient to reinstate the Th17 population and induce arthritis.

#### CIA model

The CIA animal model is created by immunization with type II collagen, which results in polyarthritis with similar clinical features to those of RA in humans [7]. Significant changes in the composition and diversity of the faecal microbiota appeared in the CIA mice before visible arthritis [33]. In addition, intestinal barrier impairment and inflammation were observed. After treatment with broad-spectrum antibiotics, a significant decrease of disease incidence and severity was noted, in addition to significantly reduced levels of inflammatory cytokines (IFN-γ, IL-1β, IL-6 and TNF-α). Interestingly, mucosal IL-17A, IL-22 and IL-23 were significantly elevated, indicating a local Th17 response activation. Liu *et al.* (2016) [34] demonstrated that *Lactobacillus salivarius* and *Lactobacillus plantarum* have a protective, anti-inflammatory role in CIA mice. Administration of *Lactobacillus* species decreased pro-inflammatory cytokines and cells (IL-17, TNF-α and Th17), increased anti-inflammatory ones (IL-10 and Treg) and lowered the incidence and severity of arthritis.

### Summary of murine models

In all the above murine models of arthritis, depletion of specific species in the gut microbiota resulted in decreased incidence of arthritis and impaired Th17 cytokine responses. These data strongly suggest that the intestinal microbiome is essential for immune activation and the development of arthritis. Several specific bacterial families (Lactobacillaceae and Lachnospiraceae), genera (*Prevotella*) and species (*P. copri*) have been identified to have an immunoregulatory role and contribute to the pathogenesis of inflammatory diseases [27, 30, 33]. Microbiome dysbiosis is speculated to induce a Th17 pattern of mucosal inflammation in genetically predisposed T cells, leading to B-cell activation and the production of autoantibodies that enter the circulation, migrating to joints and contributing to the development of inflammatory joint disease [30, 33].

### Critique of murine models

Murine models are an important tool for revealing causal relationships between the microbiome and pathogenesis of disease; however, there are some disadvantages. First, direct comparison between murine and human microbiota, in addition to features of arthritis, might be insufficient. A substantial number of human bacterial taxa might fail to colonize the animal gut [35], and the environmental factors affecting the human microbiome are not captured in murine models [36]. Second, most studies used a broad spectrum of antibiotics (ampicillin, neomycin, vancomycin and metronidazole) that might not guarantee totally GF conditions. Third, in most studies the induction of disease relied on the administration of an injection of fungi or bacteria, and disease development was not spontaneous. Therefore, results from murine studies need to be interpreted with caution, and clinical studies are necessary to draw conclusions.

### Clinical evidence

Sixteen clinical studies were identified to investigate the role of the microbiome in RA using 16S rRNA and shotgun metagenomic sequencing in both early-onset and established patients. The results are numerous, complex and not consistent. Supplementary Data S1, available at *Rheumatology Advances in Practice* online, illustrates the results of the studies in detail, and Table 2 presents a summarized version for the purpose of this review that focuses on the roles of *P. copri*, *Collinsella* and *Lactobacillus* in RA. The potential of the microbiome as a diagnostic and prognostic biomarker is also discussed.

**Table 2.** Summarized clinical evidence results

Author	Cases	Controls	Results
Kishikawa *et al.* (2020) [37]	*n* = 82, 71% untreated	*n* = 42, healthy	↑ *Prevotella* genus
Mena-Vazquez *et al.* (2020) [38]	*n* = 40, established	*n* = 40, healthy	↑ *Collinsella* genus, *Collinsella aerofaciens*
Alpizar-Rodriguez *et al.* (2019) [39]	*n* = 83, preclinical patients	*n* = 50, healthy first-degree relatives	↑ *Prevotella* genus, *Prevotella copri* ↑ *Lactobacillus* genus
Jeong *et al.* (2019) [40]	*n* = 29, early, untreated	*n* = 25, healthy	↑ *Prevotella* genus ↓ *Collinsella* genus
Sun *et al.* (2019) [41]	*n* = 66, established	*n* = 60, healthy	↓ *Lactobacillus* and *Alloprevotella* genera *Alloprovetella* positively correlated with: ESR and CRP
Chiang *et al.* (2019) [42]	*n* = 138, established	*n* = 21, healthy	↑ *Akkermansia* genus In RA patients with high levels of TNF-α or IL-17A compared with control: ↑ *Gammaproteobacteria* phylum ↑ *Enterobacteriaceae* and *Klebsiella* ↓ *Bifidobacterium* In RA-active patients *vs* RA-inactive patients: ↑ *Collinsela* and *Akkermansia* genera Positive correlations: *Euryarchaeota* phylum and IL-6 *Euryarchaeota* phylum and IL-17
Picchianti-Diamanti *et al.* (2018) [43]	*n* = 42, all *n* = 11, naive *n* = 31, treated	*n* = 10, healthy	In RA patients compared with controls: ↑ *Bacilli* class and *Lactobacillales* order ↓ *Faecalibacterium* genus and *Faecalibacterium prausnitzii* species Positive correlations: RF and ACPA positivity and *Roseburia* ESR and *Roseburia faecis* Negative correlations: RF and ACPA positivity and: *Bacilli* class or *Lactobacillales* genus
Forbes *et al.* (2018) [44]	*n* = 21, established	*n* = 23, healthy	↑ *Actinomyces* and *Eggerthella* genera ↓ *Roseburia* genus
Breban *et al.* (2017) [45]	*n* = 17, established *n* = 17, early, untreated	*n* = 51, healthy	In RA patients compared with controls: ↓ families Prevotellaceae, Paraprevotellaceae and Bifidobacteriaceae In early RA patients without treatment compared with controls: ↑ *Lactobacillus* species
Maeda *et al.* (2016) [27]	*n* = 17, early, untreated	*n* = 14, healthy	↑ *Prevotella copri*
Chen *et al.* (2016) [46]	*n* = 40, established	*n* = 32, all *n* = 15, healthy first-degree relatives *n* = 17, healthy unrelated	↑ genera: *Eggerthella*, *Actinomyces* and *Collinsella* ↑ Actinobacteria phylum ↓ *Faecalibacterium* genus
Zhang *et al.* (2015) [47]	*n* = 98, all *n* = 77, naive *n* = 21, DMARD treatment	*n* = 97, all *n* = 80, healthy first-degree relatives *n* = 17, healthy unrelated	In RA patients compared with controls: ↑ species: *Eggerthella lenta*, *Lachnospiraceae bacterium*, *Bifidobacterium dentium* and *Lactobacillus* ↓ species: *Haemophilus*
Scher *et al.* (2013) [48]	*n* = 70, all *n* = 44, early, untreated *n* = 26, established	*n* = 28, healthy	In early RA patients compared with control: ↑ *Prevotella copri*
Liu *et al.* (2013) [49]	*n* = 15, early, untreated	*n* = 15, healthy	↑ *Lactobacillus* genus ↑ *Lactobacilli* diversity

For detailed results, see Supplementary Data S1, available at *Rheumatology Advances in Practice* online.

### Prevotella copri

Findings associating RA with dysbiotic gut states are numerous and not consistent across the literature. However, a common finding among studies is an increase in the *Prevotella* genus and, specifically, in *P. copri* in early RA patients compared with healthy controls [27, 37, 39, 40, 48]. The overabundance of *P. copri* is not found in treated, established patients [38, 41–47]. Furthermore, by using metagenome-wide shotgun sequencing, Kishikawa et al. [37] demonstrated that multiple *Prevotella* species other than *P. copri* are increased in the gut microbiome of RA patients. Suggested mechanisms by which *P. copri* contributes to RA pathogenesis include the induction of inflammatory responses and molecular mimicry between microbial and host epitopes [3]. As discussed earlier in this review, the monocolonization of *P. copri* in GF SKG mice induced arthritis and increased Th17, IL-23 and IL-17 [27]. Also, SKG mice colonized with *Prevotella*-dominated microbiota from RA patients demonstrated an increase in the severity of arthritis, in addition to Th17 cells and Th17-related cytokines [27]. Taken together, these data suggest that dysbiosis precedes arthritis and that *Prevotella* species, and especially *P. copri*, trigger its development [25].

The pro-inflammatory role of *P. copri* is supported beyond mouse models. Pianta *et al.* (2017) [50] identified a *P. copri*-derived peptide that bound to HLA-DR molecules and induced Th1-type inflammatory responses in early RA patients. IgA and IgG antibodies against *P. copri* were also identified in both early and established patients. *Prevotella copri* antibodies were correlated with levels of Th17 cytokines and ACPAs. Moreover, *P. copri* 16S rDNA was found in the synovial fluid of a subgroup of patients. Although these findings support that the gut microbiome might induce autoimmune responses affecting joints via microbial peptides, the authors did not show correlations between intestinal *P. copri* and their derived proteins. *Prevotella copri* antigens are structurally similar to *N*-acetylglucosamine-6-sulfatase, which is an RA-citrullinated autoantigen that induces T- and B-cell responses in about half of RA patients [50]. Pianta *et al.* (2017) [50, 51] speculated that in genetically predisposed individuals, recognition of *Prevotella*-derived epitopes results in intestinal T-cell activation, which then migrates to joints. This finding suggests that *P. copri* might contribute to RA pathogenesis via molecular mimicry [20]. However, some *Prevotella* species have been shown to attenuate arthritis in murine models; specifically, *Prevotella histicola* was shown to decrease the severity of arthritis in CIA mice [52], and several other studies have shown that the genus *Prevotella* is one of the most abundant commensal bacteria in healthy individuals and exhibits properties beneficial for the host [25]. These contradictory findings highlight the role of other bacteria and genetic factors in the pathogenesis of RA. Further longitudinal studies are required to assess the contribution of *Prevotella* species in autoantibody production and the pathogenesis of RA.

### Collinsela

Several other bacterial genera have also been associated with human RA, including *Collinsella* [40, 38, 42, 46], *Lactobacillus* [43, 45, 49], *Eggerthella* [44, 46, 47] and *Actinomyces* [44, 46]. This highlights that a complex interplay between many species, in addition to genetic and other environmental factors, might contribute to the pathogenesis of inflammatory arthritis. Of those genera, *Collinsella*, and specifically the species *Collinsella aerofaciens*, was shown to increase intestinal permeability in RA murine models by reducing the expression of tight junction proteins [46]. *Collinsela aerofaciens* increased several inflammatory chemokines (IL-17A, CXCL1, CXCL5 and NF-κB1), in addition to the incidence and the severity of arthritis. These findings suggest that the induction of intestinal permeability is another potential mechanism by which gut dysbiosis contributes to RA [3]. It is suggested that the expansion of *C. aerofaciens* causes intestinal inflammation and loss of epithelial barrier integrity, which, in turn, allows the translocation of bacterial antigens into the systemic circulation and leads to activation of immune responses in distal sites, such as joints. Furthermore, studies have demonstrated a decrease in the genera *Roseburia* [44] and *Faecalibacterium* [43, 46] in RA patients. These butyrate-producing bacteria have been demonstrated to have anti-inflammatory properties and to maintain the intestinal epithelial barrier, underpinning the proposed mechanism that loss of intestinal integrity contributes to the pathogenesis of RA and indicating potential therapeutic pathways [44]. Indeed, in CIA mouse models butyrate decreased arthritis symptoms [53, 54]. However, butyrate was also shown to induce IL-23 activation, leading to Th17 cell differentiation, implying a potential role in RA pathogenesis. Further studies are needed to confirm intestinal integrity as a relevant therapeutic target for RA, in addition to the role of butyrate-producing bacteria.

### Lactobacillus

Proliferation of the genus *Lactobacillus* was reported in early RA patients [39, 45, 49] and in established patients [43, 47], whereas Sun *et al*. (2019) [41] reported a decrease of *Lactobacillus* in established patients. Zhang *et al.* (2015) [47] showed that *L. salivarius* (oral and intestinal) was correlated with disease activity. The correlation of *Lactobacillus* with RA is in accordance with murine studies. Monocolonization with *L. bifidus* was sufficient to induce arthritis in IL-1ra^−/−^ mice [29], and overabundance of the genus *Lactobacillus* was reported in CIA mice before the development of arthritis [33]. In a similar manner to *Prevotella*, it has been suggested that *Lactobacillus* species might contribute to RA pathogenesis via increasing Th17 cells and Th17-related cytokines and activating Th1-cell responses [3]. However, several murine and human studies have shown that oral administration of *Lactobacillus* species led to amelioration of arthritis and reduction of inflammation [39]. A recent study showed that the treatment of rats with *Lactobacillus casei* restored gut dysbiosis and decreased arthritis severity and pro-inflammatory cytokines [55]. *Lactobacillus salivarius* and *L. plantarum* reduced Th17 cells and increased Treg in CIA mice, resulting in reduced arthritis severity [34]. A systematic review concluded that supplementation with *Lactobacillus* species leads to decreased IL-6 but does not significantly change arthritis in humans [56]. Therefore, the results on *Lactobacillus* species need to be interpreted with caution, and further studies are necessary, especially given that *Lactobacillus* species are frequently administered as probiotics.

#### The microbiome as a diagnostic and prognostic biomarker

The potential of the microbiome as a diagnostic and severity biomarker is supported by several clinical studies revealing correlations between certain bacteria and clinical parameters of RA (see Table 2). Sun *et al.* (2019) [41] found that *Alloprevotella* was positively correlated with RF, ESR and CRP. In the study by Chiang *et al.* (2019) [42], the inflammatory markers TNF-α and IL-17A were positively correlated with the Gammaproteobacteria phylum, Enterobacteriaceae and *Klebsiella*, and negatively correlated with *Bifidobacterium*, while the genera *Collinsela* and *Akkermansia* were positively correlated with disease activity. Disease activity has also been positively correlated with phylum Euryarchaeota, and phylum Euryarchaeota has been proposed as an independent risk factor by multivariate analysis [43]. Zhang *et al.* (2015) [47] showed that *Haemophilus* species are negatively correlated with the levels of serum antibodies. Research on the topic is limited, but larger longitudinal and metagenome-wide association studies can play a central role in determining microbial biomarkers that will allow early diagnosis and therapeutic interventions.

#### Summary and critique of clinical evidence

As illustrated in Supplementary Data S1, available at *Rheumatology Advances in Practice* online, the results from the clinical studies are extensive and not consistent, emphasizing the complexity of the subject. A distinct RA microbiome is difficult to prove, especially given that studies so far have been heterogeneous and failed to take into consideration environmental and lifestyle factors. Differences among studies include: differences in disease (duration, activity, genetic and inflammatory markers and treatment), differences in patient characteristics (race, age, sex, BMI and socioeconomic status), and differences in environmental and lifestyle factors (diet, smoking, physical activity and geographical location) [3]. However, all studies demonstrate alterations in the gut microbiome in both early and established patients, and it can therefore be concluded that gut dysbiosis plays a central role in RA. This review has focused on the most repetitive findings: overabundance of *P. copri* in early onset of RA, and proliferation of the genera *Collinsella* and *Lactobacillus*. Three potential mechanisms by which microbiota might contribute to RA pathogenesis have been discussed: inflammatory responses (*P. copri* and *Lactobacillus*), molecular mimicry (*P. copri*) and loss of integrity of the intestinal barrier (*Collinsella*). It is worth noting that evidence for increased intestinal permeability in RA is poor and potentially biased, owing to frequent use of NSAIDs that are well associated with enteropathy [21]. The protective but non-conclusive role of butyrate-producing bacteria (*Roseburia* and *Faecalibacterium*) has also been discussed. Longitudinal studies with multiple statistical methods to control confounders are necessary in order to identify characteristic microbial alterations, to comprehend the mechanisms involved and to determine diagnostic and prognostic biomarkers to reveal the therapeutic potential of the microbiome.

## Future considerations: microbiome manipulation

The arthritogenic, inflammatory role of some bacteria and the protective, anti-inflammatory role of others implies that microbiome manipulation might be an effective treatment or a preventive intervention for genetically predisposed individuals. Three therapeutic options for manipulating the microbiome are proposed: diet, probiotic supplementation and faecal microbiota transplantation.

### Diet

Research on how diet manipulates the microbiome and affects RA is limited and difficult, owing to complex confounding factors. However, high-fibre and healthy diets have been shown to have anti-inflammatory effects, whereas poor diets rich in saturated fats increase intestinal permeability and inflammation [57, 58].

### Probiotics

It has already been discussed in this review that *L. casei* supplementation was shown to decrease arthritis in mice and to improve clinical and inflammatory biomarkers in human RA patients [39, 55]. To date, studies exploring the efficacy and effectiveness of supplementation with probiotics and dietary interventions in restoring gut dysbiosis and aiding in the management and prevention of RA are limited.

### Faecal microbiota transplantation

Faecal microbiota transplantation has been shown to be an efficient treatment for intestinal disease. If the intestine is indeed the initial site where immune changes take place, faecal microbiota transplantation is a potential treatment and preventive method for inflammatory joint disease. To our knowledge, there is only one randomized controlled trial assessing the efficacy and safety of faecal microbiota transplantation on PsA patients, but the results are not yet published [59].

Diet, probiotic supplementation and faecal microbiota transplantation are minimally invasive procedures with relatively few side effects and could therefore provide appealing treatment options for patients. Randomized controlled trials are needed to explore the effectiveness and safety of these treatments.

## Conclusion

Research on the effect of the microbiome on inflammatory joint disease is complex and difficult, owing to many confounding factors. However, murine models of arthritis strongly suggesting that gut dysbiosis precedes arthritis and that local intestinal inflammation might lead to systemic inflammation in genetically predisposed individuals. Although a characteristic RA microbiome has not been identified, clinical studies suggest that overabundance of *P. copri* in early patients contributes to the pathogenesis of RA by inducing inflammatory responses and molecular mimicry, while overabundance of the genus *Collinsela* might induce inflammatory joint disease by increasing intestinal permeability that allows translocation of inflammatory cytokines to joints. Some *Lactobacillus* species (*L. salivarius* and *L. bifidus*) induce inflammation and are associated with increased incidence of arthritis, whereas other *Lactobacillus* species reduce inflammation and arthritis symptoms. Recent studies are unfolding the potential of the microbiome as a diagnostic and severity biomarker that will allow early diagnosis and determine therapeutic interventions, such as diet, probiotics and faecal microbiota transplantation.

## Supplementary Material

rkad034_Supplementary_Data

## Data Availability

No new data were generated in support of this article.
