# Supplementary material for: The role of the microbiome in rheumatoid arthritis: a review
Source: Rheumatol Adv Pract. 2023 Apr 3;7(2):rkad034. doi: 10.1093/rap/rkad034 (PMC11007908; doi:10.1093/rap/rkad034)
Supplement: rkad034_Supplementary_Data [file rkad034_supplementary_data.docx]

**Supplementary Material**

**Supplementary Data S1: PubMed Search strategy**

The full search strategy for PubMed was the following: ((("Gastrointestinal Microbiome"[Mesh]) OR ("gastrointestinal microbiome*"[Title/Abstract] OR "gut microbiome*"[Title/Abstract] OR "gut microflora*"[Title/Abstract] OR "gut microbiota*"[Title/Abstract] OR "gastrointestinal flora*"[Title/Abstract] OR "gastrointestinal microbiota*"[Title/Abstract] OR "gut flora*"[Title/Abstract] OR "gastrointestinal microbial communit*"[Title/Abstract] OR "gastrointestinal microflora*"[Title/Abstract] OR "intestinal microbiome*"[Title/Abstract] OR "intestinal microbiota*"[Title/Abstract] OR "intestinal flora*"[Title/Abstract] OR "enteric bacteria"[Title/Abstract])) AND ((("Joint Diseases"[Mesh]) OR ("joint disease*"[Title/Abstract] OR arthritis[Title/Abstract] OR arthopath*[Title/Abstract] OR rheumat*[Title/Abstract] OR "ankylosing spondylitis"[Title/Abstract]))) AND ((english[Filter]) AND (2010:2021[pdat]))].

**Supplementary Data S1: Clinical evidence studies results**

| **Author** | **Methodology** | **Cases** | **Control** | **Results** |
| --- | --- | --- | --- | --- |
| Kishikawa 2020  (34) | Shotgun metagenomic sequencing | n=82,  71%  Untreated | n=42, Healthy | In RA patients compared to control:  ↑ *Prevotella* genus  ↓ gene ID: R6FCZ7  ↑ biological pathways related to metabolism  No difference in diversity |
| Mena-Vazquez  2020 (38) | 16S rRNA sequencing | n=40, Established patients on  DMARDs treatment | n=40, Healthy | In RA patients compared to control:  ↑ genera:   - *Collinsella* - *Sedimentibacter* - Enterococcus   ↑ *Collinsella* *aerofaciens* species ↓ genera:   - *Sarcina* - *Porphyromonas*   ↓ *Dorea* *formicigenerans* species ↓ β diversity |
| Alpizar-Rodriguez  2019 (35) | 16S rRNA sequencing | n=83,  Pre-clinical patients | n=50, Healthy first-degree relatives | In RA patients compared to control: ↑ genera   - *Prevotella* - *Lactobacillus*   ↑ *Prevotella* species, *Prevotella* *copri*  No difference in α and β diversities |
| Lee 2019 (60) | 16S rRNA sequencing | n=9,  Established | n=9,  Patients with osteoarthritis | In RA patients compared to OA:  lower *Bacteroidetes*: *Firmicutes* ratio  ↓ certain species:   - *Fusicatenibacter saccharivorans* - *Dialister invisus* - *Clostridium leptum* - *Ruthenibacterium lactatiformans* - *Anaerotruncus colihominis* - *Bacteroides faecichinchillae* - *Harryflintia acetispora* - *Bacteroides acidifaciens* - *Christensenella minuta*   No difference in diversity |
| Jeong 2019 (36) | 16S rRNA sequencing | n=29, Early, untreated | n=25, Healthy | In RA patients compared to control:  ↑ *Bacteroidales* order  ↑ *Prevotella* genus  ↓ *Collinsella* genus ↓ α diversity |

| **Author** | **Methodology** | **Cases** | **Control** | **Results** |  |
| --- | --- | --- | --- | --- | --- |
| Muñiz Pedrogo  2019 (61) | Shotgun metagenomic sequencing | n=25,  Established | n=64, Healthy | In RA patients compared to control:  ↑ *Clostridiaceae* family ↑ Proteobacteria phylum:  ↑ *Epsilonproteobacteria* class  ↑ *Campylobacterales* class No difference in diversity |  |
| Sun 2019 (39) | 16S rRNA sequencing | n=66,  Established | n=60, Healthy | In RA patients compared to control: ↑ genera   - Bacteroides - *Escherichia-Shigella*   ↓ genera:   - *Lactobacillus* - *Enterobacter* - *Alloprevotella* - *Odoribacter*   ↓ α and β diversity    Alloprovetella positively correlated with:  Rheumatoid factor Inflammatory markers:   - ESR - CRP |  |
| Chiang 2019 (40) | 16S rRNA sequencing | n=138,  Established | n=21, Healthy | In RA patients compared to control: |  |
|  |  |  |  | ↑ *Verrucomicrobia* phylum  ↑ *Akkermansia* genus    In RF-negative RA patients compared to control: |  |
|  |  |  |  | ↓ α diversity    In ACPA-positive RA patients compared to control: |  |
|  |  |  |  | ↓ α diversity  In RA patients with high levels of TNF-α or IL17A |  |
|  |  |  |  | In RA active patients versus RA inactive patients:  ↑ *Collinsela* and *Akkermansia* genera  In RF-positive RA patients compared to RF-negative patients:  ↑ *Blautia* and *Collinsela* genera  Positive correlations:  *Euryarchaeota* phylum and IL-6  *Euryarchaeota* phylum and IL-17  *Tenericutes* phylum and IL-6  *Tenericutes* phylum and IL-17α |  |
|  |  |  |  |  |  |
|  |  |  |  |  |  |

| **Author** | **Methodology** | **Cases** | **Control** | **Results** |  |
| --- | --- | --- | --- | --- | --- |
| Picchianti-Diamanti  2018 (41) | 16S rRNA sequencing | n=42, All n=11, naive n=31, treated | n=10, Healthy | In RA patients compared to control: |  |
|  |  |  |  | ↑ *Bacilli* class and *Lactobacillales* order  ↓ *Faecalibacterium* genus and *Faecalibacterium* prausnitzii species  ↓ *Flavobacterium* genus and *Blautia* *coccoides* species  No difference in α and β diversity  RA patients receiving ETN treatment compared to naive RA patients:  ↑ *Cyanobacteria*  ↑ *Nostocophycidae* class and *Nostocales* order ↓ *Deltaproteobacteria* class and *Clostridiaceae* family  RA patients receiving MTX treatment compared to naive RA patients:  ↓ *Enterobacteriales* order  Positive correlations:  RF and ACPA positivity and:   - *Roseburia* ESR and: - *Enterobacteriales* order - *Roseburia* *faecis* - *Streptococcus* *parasanguinis*   CRP and:   - *Parabacteroides distasonis*   Negative correlations:  RF and ACPA positivity and:   - *Bacilli* class - *Lactobacillales* genus - *Streptococcus* *vestibularis* |  |

| **Author** | **Methodology** | **Cases** | **Control** | **Results** |  |
| --- | --- | --- | --- | --- | --- |
| Forbes 2018 (42) | 16S rRNA sequencing | n=21,  Established | n=23, Healthy | In RA patients compared to control:  ↑ genera:   - *Actinomyces* - *Eggerthella* - *Clostridium III* - *Faecalicoccus* - *Streptococcus*   ↓ genera   - *Gemmiger* - *Lachnospira* - *Sporobacter* - *Roseburia* |  |
| Breban 2017 (43) | 16S rRNA sequencing | n=17,  Established    n=17, Early, untreated | n=51, Healthy | In RA patients compared to control: |  |
|  |  |  |  | ↑ *Proteobacteria* phylum ↓ families:   - *Prevotellaceae* - *Paraprevotellaceae* - *Bifidobacteriaceae*   ↓ α diversity    In early RA patients without treatment compared to |  |
|  |  |  |  | control:  ↑ *Lactobacillus* species  ↑ *Corynebacterium* variabile  ↑ *Staphylococcus* *aureus*  ↑ *Facklamia*  ↑ *Paraprevotallaceae* family |  |
|  |  |  |  |  |  |
| Maeda 2016 (26) | 16S rRNA sequencing | n=17, Early, untreated | n=14, Healthy | In early RA patients compared to control:  ↑ *Prevotella copri* |  |
| Chen 2016 (44) | 16S rRNA sequencing | n=40,  Established | n=32, All n=15, Healthy first degree relatives n=17, Healthy unrelated | In RA patients compared to control:  ↑ genera:   - *Eggerthella* - *Actinomyces* - *Collinsella*   ↑ *Actinobacteria* phylum  ↓ genera:   - *Faecalibacterium*   ↓ diversity  No association with *Prevotella copri* |  |
| **Author** | **Methodology** | **Cases** | **Control** | **Results** |  |
| Zhang 2015 (45) | Shotgun metagenomic sequencing | n=98, All n=77, Naive n=21,  DMARDs treatment | n=97, All n=80, Healthy first degree relatives n=17, Healthy unrelated | In RA patients compared to control:  ↑ *Proteobacteria* phylum ↑ species:   - *Clostridium asparagiforme* - *Gordonibacter pamelaeae* - *Eggerthella lenta* - *Lachnospiraceae bacterium* - *Bifidobacterium dentium* - *Lactobacillus* - *Ruminococcus lactaris*   ↓ *Firmicutes* phylum  ↓ species:   - *Veillonella* - *Haemophilus* - *Klebsiella pneumoniae* - *Bifidobacterium bifidum* - *Sutterella wadsworthensis* - *Megamonas hypermegale*   No difference in diversity  DMARD treatment partially restores dysbiosis |  |
| Scher 2013 (37) | 16S rRNA sequencing    Shotgun metagenomic sequencing in subgroup | n=70, All n=44, Early, untreated n=26, Established | n=28, Healthy | In early RA patients compared to control:  ↑ *Prevotella copri*  ↓ *Bacteroides* species  ↓ *Clostridia* class  ↓ *Lachnospiraceae* family    In RA patients compared to control:  No difference in diversity |  |
| Liu 2013 (48) | 16S rRNA sequencing | n=15, early, untreated | n=15, Healthy | In RA patients compared to control:  ↑ *Lactobacillus* genus  ↑ *Lactobacilli* diversity |  |

ESR: erythrocyte sedimentation rate, CRP: C-reactive protein, ETN: etanercept, MTX: methotrexate, RF: rheumatoid factor, ACPA: anti-citrullinated protein antibody, DMARDs: Disease-modifying anti-rheumatic drugs.
